# Top-down and bottom-up forces explain patch utilization by two deer species and forest recruitment

**DOI:** 10.1007/s00442-022-05292-8

**Published:** 2022-11-24

**Authors:** J. Ignacio Ramirez, Lourens Poorter, Patrick A. Jansen, Jan den Ouden, Matthias Siewert, Johan Olofsson

**Affiliations:** 1 Department of Ecology and Environmental Sciences, Umeå University, Umeå, Sweden; 2 Forest Ecology and Forest Management Group, Wageningen University and Research, Wageningen, The Netherlands; 3 Wildlife Ecology and Conservation Group, Wageningen University and Research, Wageningen, The Netherlands; 4 Smithsonian Tropical Research Institute, Balboa, Ancon, Panama

**Keywords:** Patch dynamics, Forest composition, Herbivory, Forest edge, Safety-in-numbers

## Abstract

Ungulates play an important role in temperate systems. Through their feeding behaviour, they can respond to vegetation by selecting patches or modify vegetation composition by herbivory. The degree in which they interact with vegetation can either reinforce landscape heterogeneity by creating disturbance or reduce heterogeneity in case of overbrowsing. This study evaluates how bottom-up (patch quality, structure), top-down forces (hunting, distance to village, forest edge) and deer features (feeding type, abundance) mediate patch utilization in a temperate forest and assess the implications of patch utilization and light on forest recruitment. Theory predicts that animals seek to maximize their energetic gains by food intake while minimizing the costs associated to foraging, such as the energy required for avoiding predators and exploiting resources. We focused on two deer species with contrasting feeding type: a browser (*C. capreolus*) and a mixed feeder (*C. elaphus*). We paired camera traps to vegetation sub-plots in ten forest sites in the Netherlands that widely ranged in deer abundance and landscape heterogeneity. Results showed that patch utilization is simultaneously explained by bottom-up, top-down forces and by deer abundance, as predicted by the safety-in-numbers hypothesis. Yet, forces best explaining patch utilization differed between deer species. Overall, higher patch utilization came with higher browsing, lower tree diversity and a large difference in forest composition: from a mix of broadleaves and conifers towards only conifers. We conclude that these two deer species, although living in the same area and belonging to the same guild, differentially perceive, interact with and shape their surrounding landscape.

**Supplementary Information:**

The online version contains supplementary material available at 10.1007/s00442-022-05292-8.

## Introduction

Wild ungulate abundance has increased substantially in the Northern hemisphere during the past decades due to a reduction in the numbers of top predators and hunting pressure, and due to an increase in conservation and rewilding initiatives (Kuiters et al. 1996; Côté et al. 2004). Through their feeding behaviour, ungulates may strongly respond to vegetation by selecting patches and modify it by herbivory (Ramirez et al. 2018, 2019, 2021a). Ungulate feeding behaviour may either reinforce landscape heterogeneity, and hence, plant and animal diversity, or reduce landscape heterogeneity, in case of overbrowsing (Gill and Beardall 2001; Ramirez et al. 2021b). The degree of patch utilization is therefore key to understand the potential effects of ungulates on the landscape and vice versa. Patch utilization is modulated by bottom-up forces (i.e., resource availability) and top-down forces (i.e., predation risk), where the relative strength of these forces depends on ecosystem conditions and ungulate characteristics (Rieucau et al. 2009). Yet, these forces are not entirely understood due to the inherent complexities of ecological systems (Vickery et al. 2011; Seidel and Boyce 2015). Here we evaluate for Dutch temperate mixed forests, how patch utilization by two deer species is driven by their features (i.e., feeding type and abundance), top-down and bottom-up forces. Additionally, we assess how patch utilization, browsing intensity and light availability explain forest recruitment composition.

Top-down forces are formed by (fear of) predation (Estes et al. 2011). Direct effects of predation refer to the number of animals preyed, and indirect effects refer to avoidance of risky areas and increased vigilance (Lima 1998). Besides apex predators, humans may also exert top-down pressures directly by hunting and indirectly by altering the structure of natural areas (Callan et al. 2013; Cromsigt et al. 2013; Gaynor et al. 2018). Overall, herbivores respond to these forces by avoiding risk in time and space, which translates to less time being in patches perceived as unsafe and shifting daily activity away from times perceived as unsafe (Kuijper et al. 2013; Gaynor et al. 2018). Herbivores may also adapt to the environment by forming large groups where they find safety-in-numbers (Hamilton 1971; Hager and Helfman 1991; Brown et al. 1999). This reduces not only the likelihood of an individual being preyed, but also by having sentinel individuals that can alarm the group in case of danger (Lima and Dill 1990; Lima 1995a; Lima and Zollner 1996). The formation of groups, however, may involve the cost of intra-specific competition for reproduction and resources (Apollonio 1989; Beauchamp and Ruxton 2003) which, in turn, may reduce the time spent in a patch by elevating energetic costs.

Bottom-up forces are related to the quality of the vegetation and structure of the patch (McNaughton et al. 1989; Fryxell 1991). Plant species composition of the patch indicates the food quantity and quality that is available for herbivores. The Marginal Value Theorem predicts that herbivores spend more time in high-quality patches to maximize energy intake rate per foraging unit (Charnov 1976). Vegetation structure refers to the vertical layering and horizontal distribution of the vegetation, which determines the degree of cover and the concentration of food an animal can find. Herbivores often spend more time in patches that offer shelter from weather and predators (Brown 1988; Brown et al. 1999).

The time herbivores spend in a patch relative to other patches is the outcome of balancing food intake and risk avoidance. This differs between species, depending on the breadth of the diet and the degree of sociality (Hofmann 1989; Gill 1992a). Among cervids, for example, browsers may spend more time in forest patches because they strictly feed on woody plants while mixed feeders—which also graze on graminoids—will spend more time in open environments where the graminoids are most abundant. Second, social cervids form larger groups than solitary cervids, and thus pose the advantage of risk dilution against predators and collective vigilance (Lima 1995b; Bednekoff and Lima 1998).

Patch utilization by cervids in turn affects the structure, composition and succession of forests in a direct way by consumption and trampling of saplings, and indirectly by seed dispersal, changing the competitive balance between plant species and nutrient input (Gill 1992a, b; Eycott et al. 2007; Murray et al. 2014). In general, palatable tree species are browsed more intensively, providing a competitive advantage for less palatable species to establish in the forest understory and eventually reach the forest canopy (Pastor and Naiman 1992; Ramirez et al. 2019). Because vegetation responds to browsing pressure in a non-linear way, shifts in vegetation composition and structure already start at low deer densities (Ramirez et al. 2021a). If persistent over time, these effects may also cascade to other trophic levels and ecosystem processes which can ultimately promote a shift of forests to alternative states (Nuttle et al. 2014; Davalos et al. 2015; Ramirez et al. 2021b).

Studies have partially addressed how patch utilization by wildlife varies with top-down (Kuijper et al. 2013; Shrader et al. 2008), bottom-up forces (Rieucau et al. 2009; Wei et al. 2015), the landscape matrix (Rieucau et al. 2007; Bubnicki et al. 2019) and population size (China et al. 2008; Ramirez et al. 2021c) but relatively few studies have analysed the relative importance of all these factors in combination. They show in agreement with the Optimal Foraging Theory that animals select patches with high quality food and with low predation risk, and their time in the patch is longer when being as a group (Brown 1988). How patch utilization differs between species with contrasting feeding preferences within the same area, remains unknown. Dependency in patch utilization is expected because species experience the environment according to their life history traits and phenotypic characteristics.

**Fig. 1:**
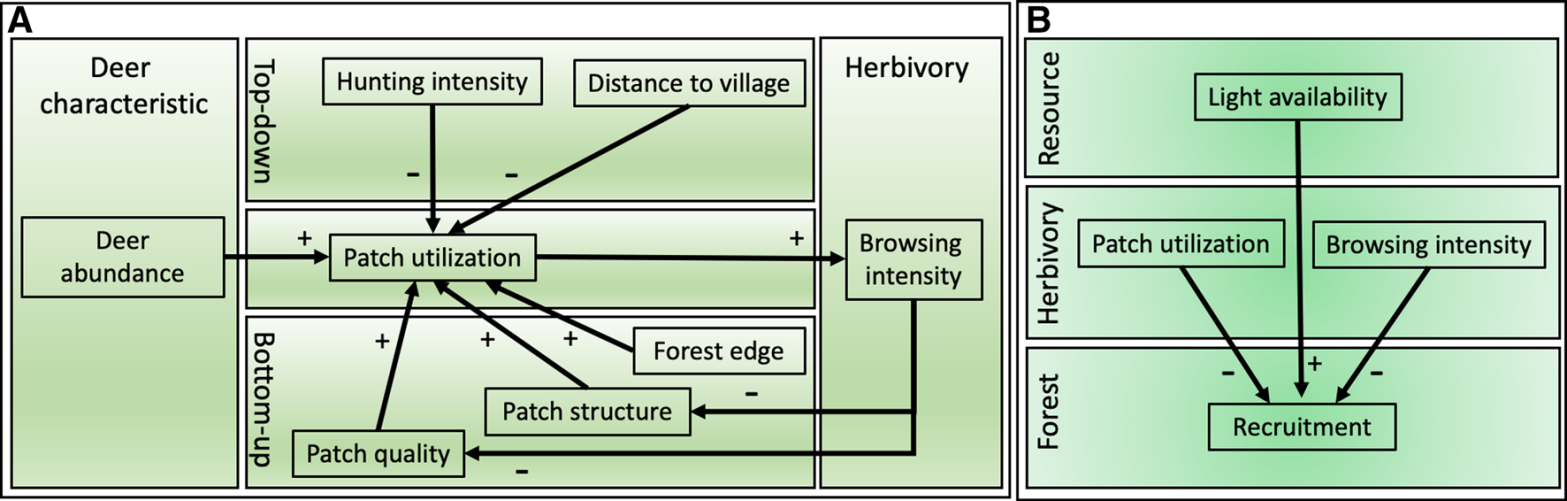
Conceptual models of how patch utilization is driven by top-down, bottom-up forces and deer characteristics (panel **A**) and a model for how forest recruitment is modulated by patch utilization, light and browsing (panel **B**). The direction of the relationship is shown with ( +) and (−) signs

This study aimed to understand the relative importance of top-down and bottom-up forces in determining patch utilization by two deer species that differ in feeding type and social behaviour. Roe deer (*Capreolus capreolus*) is a browser and a solitary species (Hofmann 1989; Kjellander et al. 2004), whereas red deer (*Cervus elaphus*) is a mixed feeder and a social species (Georgii 1981; Hofmann 1989). Our approach was to pair camera traps to vegetation sub-plots in ten Dutch forests (Smith et al. 2020) and quantify patch utilization, top-down (distance to village and forest edge, hunting intensity, Fig. 1), bottom-up drivers (vegetation composition, structure) and deer features (feeding type, and abundance). We asked the following questions: (I) which factors explain patch utilization by deer? (II) Does this differ between species? (III) How does patch utilization, browsing intensity and light availability relate to tree species composition? We predict that (i) patch utilization is best explained by bottom-up and top-down forces because cervids are often culled in these forests. (ii) This is stronger for the strict browser (roe deer) than for the mixed feeder (red deer) which can find safety-in-numbers. (iii) Vegetation composition is best explained by browsing intensity and light availably since these are the limiting factor in temperate forests and are directly related to plant performance and vegetation composition.

## Materials and methods

### Research location

**Fig. 2:**
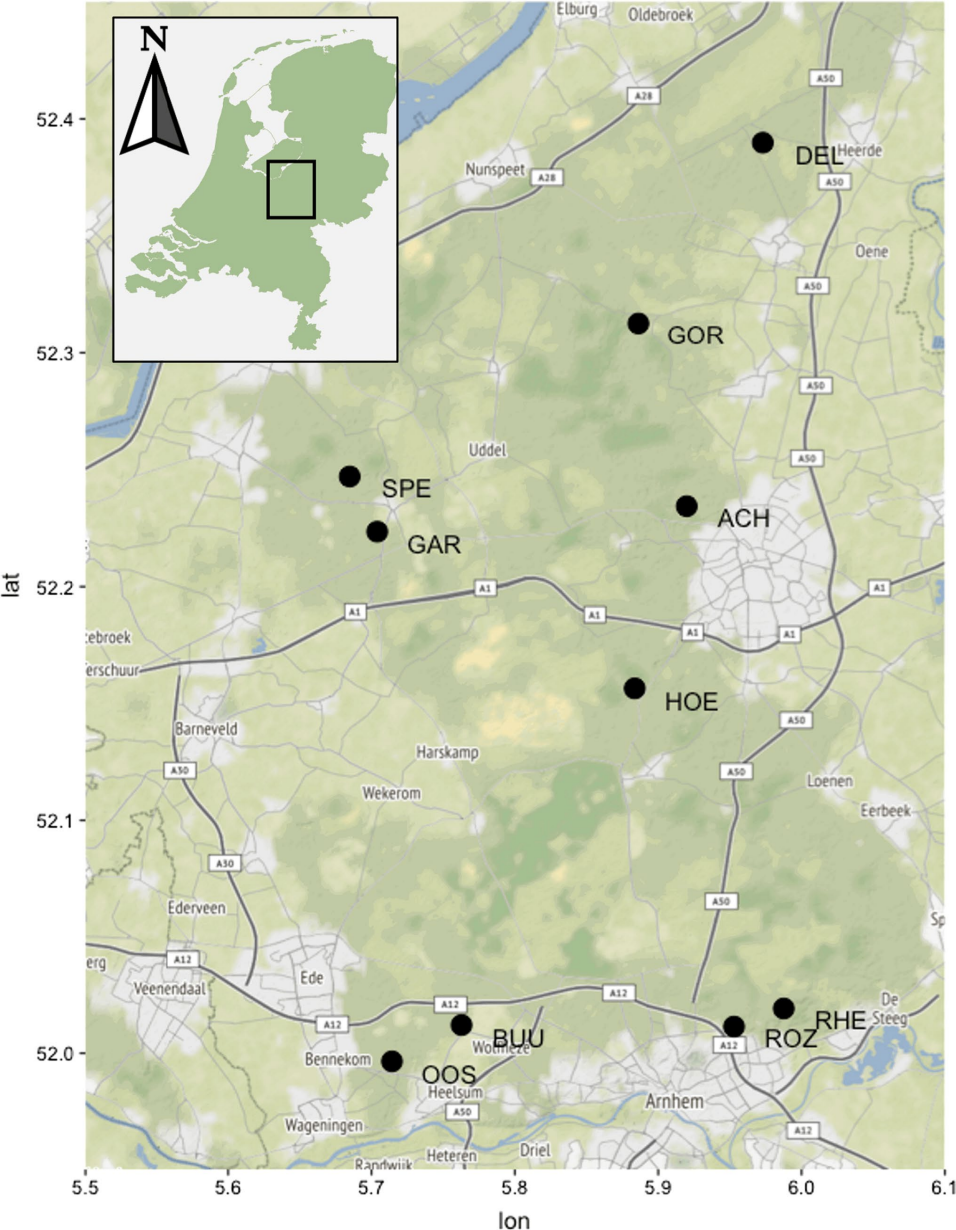
Research sites in the Veluwe, the Netherlands. Black dots and codes indicate research sites, dark green forest cover, light green non-forest areas and light grey urban build-up. Oostereng (OOS), Buunderkamp (BUU), Speulderbos (SPE), Gortel (GOR), Hoenderloo (HOE), Dellen (DEL), Garderen (GAR), Achterpark (ACH), Rozendaalse (ROZ) and Rheden (RHE). Size of map is 55 × 40 km

Research was conducted in the Veluwe, a 1100-km^2^ forest region located in the central part of the Netherlands (52° 11′ 42′′ N 5° 50′ 57′′ E, Fig. 2). The annual average temperature is 10.5 °C and average rainfall is 850 mm y^−1^. Soils are sandy with varying textures and loam content, ranging from extremely dry and nutrient-poor entisols to xeric humic podzols, and brown earths (inceptisols) on the more loam-rich sands (Kuiters and Slim 2002). Two third of the area is covered by a mix of broadleaf and conifer species and the remaining third consists of heathland, grassland and dunes. The main broadleaf species are birch (*Betula pendula*), oak (*Quercus robur*), red oak (*Quercus rubra*) and beech (*Fagus sylvatica*); the main conifer species are scots pine (*Pinus sylvestris*) and the non-natives spruce (*Picea abies*), Douglas fir (*Pseudotsuga menziesii*) and larch (*Larix kaempfer*i) (Ramirez et al. 2019). The ungulate assemblage is composed by red deer (*Cervus elaphus*), roe deer (*Capreolus capreolus*), wild boar (*Sus scrofa*), a few fallow deer (*Dama dama*) and introduced mouflon (*Ovis orientalis*). At time of the research, the area lacked top predators for more than a century, but experienced high hunting pressure. The area is intersected by a wide network of highways, roads, train tracks and towns, dividing the Veluwe in several compartments that vary widely in deer abundance and forest heterogeneity. The Veluwe contains several fields surrounded by forests and is intensely used as a recreational area.

### Sampling design

We selected ten tracts of continuous forest across the Veluwe, such that these ranged widely in deer population densities and in physical attributes (Table 1). In each forest, we selected square plots of 1 km^2^ using ArcGIS. The minimum distance between plots was 5 km, sufficient to minimize deer home range (roe deer, 0.1–1.5 km^2^, red deer 5–10 km^2^) overlap (Gill and Morgan 2010). Within each plot, 21 random points with at least 100 m of interspacing were generated. At each point, a Reconyx HyperFire HC500 camera trap was mounted to the closest tree at 50 cm height and facing north. Vegetation 3 m in front of the cameras was cut to below 50 cm to maximize detection by the cameras. Maximum detection area was then quantified using the walk test: walking away from the cameras and measuring the distance to the furthest point where the cameras detected movement and quantifying the detection area with the camera lens angle (42°). These values were used to correct for variation in detection area between camera traps. We deployed three camera traps sequentially for each site, cameras were collected and moved to three new random points after approximately 21 days (± 4.8 days). This procedure was repeated until all random points were surveyed. This yielded an average of 463 camera trap days per forest site (02 June–27 October 2017). We assume no variation in seasonal behaviour of deer across sites since camera surveys were conducted sequentially and simultaneously across all sites. All images were grouped into sequences that represented different events and annotated by using the software “Agouti” (Casaer et al. 2019). Species identity, number of individuals, and time spent in the forest patch were recorded. For all sequences with deer, we recorded whether animals were feeding. Browsing incidence was defined as the number of times an animal lowered the head to the ground in proportion to the camera detection area and number of deployment days (obs m^−2^ d^−1^).

**Table 1:** Characterization of the ten forest sites in the Veluwe, the Netherlands

F. site	Top-down		Bottom-up			Abundance		Patch utilization		Abiotic	Browsing	
	Hunt	Town	P. quality	P. structure	F. edge	Roe	Red	Roe	Red	Light	Roe	Red
ACH	4.10	4.1	72.57	21.36	0.67	0.09	0.11	0.08	0.02	35.41	0.004	0.000
BUU	3.83	7.2	71.81	20.50	0.68	0.26	0.25	0.24	0.17	36.15	0.005	0.004
DEL	7.72	16.0	47.45	22.91	0.16	0.11	0.29	0.09	0.16	37.09	0.004	0.003
GAR	4.10	18.0	89.43	27.79	0.21	0.14	0.11	0.11	0.02	37.34	0.004	0.000
GOR	0.01	13.1	98.95	23.50	0.30	0.18	2.83	0.17	2.78	37.84	0.002	0.015
HOE	5.84	8.0	75.18	26.05	0.22	0.09	0.29	0.05	0.24	39.92	0.002	0.006
OOS	0.13	5.8	71.52	23.14	0.15	0.22	0.00	0.21	0.00	40.91	0.004	0.000
RHE	9.48	7.4	87.24	22.57	0.90	0.14	0.28	0.12	0.19	39.57	0.004	0.004
ROZ	9.48	5.0	70.67	30.29	0.54	0.04	0.25	0.03	0.15	41.70	0.001	0.002
SPE	4.10	19.4	47.14	25.86	0.25	0.21	0.06	0.20	0.03	42.39	0.013	0.001

Average values of the 21 sapling points for each site are provided. Units are expressed as follows: hunting intensity (ind km^−2^ y^−1^), distance to town (km), patch quality (broadleaf %), patch structure (basal area in m^2^ ha^−1^), distance to forest edge (km), landscape abundance of deer (ind m^−2^ d^−1^), patch utilization by deer (ind s m^−2^ d^−1^), light availability in the understory (%) and browsing intensity (obs m^−2^ d^−1^)

Vegetation sub-plots were set up 3 m away from the cameras, with a fixed width of 4 m and a variable length, large enough to include 50 stems between 10 and 250 cm in height. All stems were identified to species, the height was measured as the distance from the ground to the apical shoot and browsing signs were inspected for the entire plant and annotated as present or absent. Diameter at breast height was measured for stems > 3 cm in diameter. Since saplings composition is dependent on light, we used a densiometer to measure light availability by standing on the centre of the vegetation plot (Lemmon 1956). The values obtained from the densiometer positively correlate to the photosynthetical active radiation in the forest understory (Baudry et al. 2014). Shrubs, herbs and ferns were also measured in the plots, but we opted not to include as it had no relationship with any of our variables. The establishment of the plots and vegetation measurements were done during the collection of the camera traps.

### Top-down, bottom-up forces and patch utilization

To quantify top-down forces, we calculated for each forest site the proximity to urban areas (which we used as an index for visiting rates) and hunting intensity (as an index for both direct and indirect effects of predation). Distance-to-town was measured as the distance from the centroid of the forest plot to the nearest large town centres (> 40,000 inhabitants), using Google Earth. Distance from camera points to towns was not included because it yielded an average value similar as to the centroid. Smaller villages are abundant in the area and hence, they were omitted because they did not provide a gradient. Hunting intensity was quantified as the take-off density (i.e., ind km^−2^ y^−1^) which was sourced from official culling data (http://www.verenigingwildbeheerveluwe.nl).

To quantify bottom-up forces, we measured the quality and the structure of the vegetation as these variables are related to food availability and shelter from predators and weather. Patch quality was measured as the percentage of broadleaf stems (the remainder being conifers %) and patch structure as the basal area of saplings (m^2^ ha^−1^). Distance from the vegetation plot to the nearest forest edge was also measured since roe deer is a browser, while red deer is a grazer (Hofmann 1989). Finally, we derived Shannon (H) diversity for all vegetation plots. To quantify landscape abundance of deer, we calculated the average trap rate per forest site (ind m^−2^ d^−1^) using the following formula:

$${\text{Trap rate}} = \mathop \sum \limits_{i = 1}^{n} \frac{A}{B \times C}$$

where (*A*) is the total number of individuals in the image sequences, (*B*) is the maximal detection area of the camera in m^2^ and (*C*) is the sampling effort in days. Patch utilization was then quantified for each individual sampling point (ind s m^−2^ d^−1^), as follows:

$${\text{Patch utilization}} = \mathop \sum \limits_{i = 1}^{n} \frac{A \times T }{{B \times C}}$$

where (*T*) is the total time that animals spent in front of the camera trap.

### Statistical analysis

All statistical analysis were done in R version 4.0.2 (R Core Team 2013). Linear Mixed Models (LMM) were employed to construct a pathway model that quantifies how top-down (hunting intensity, distance to town), bottom-up (distance to forest edge, patch quality, structure) forces and deer characteristics (feeding type, abundance) explained variation in patch utilization, and how patch utilization feeds back to vegetation composition and structure (Ramirez et al. 2021b). We tested for collinearity before the formal analysis and results indicated independence among factors. Six independent models were conducted. For three of the models, patch utilization was the response, forest site was a random factor to account for the nested design, and top-down, bottom-up, and deer characteristics, respectively, were independent fixed factors. The fourth model looked at the relationship between browsing intensity and patch utilization, with forest site as a random factor. The fifth and sixth models, tested how browsing intensity feeds back to vegetation composition and structure, with forest site as a random factor. For each cervid species separate models were made. A *p* value < 0.05 indicated a significant relationship between response and fixed factors and all given coefficients were standardized. A priori and using LMM, we evaluated and found that red and roe deer did not interfere with each other’s patch utilization (Electronic Supplemental Material A1). The package “lme4, version 1.1–23” was used for modelling (Bates et al. 2015) and the package “ggplot2, version 3.3.2” for plotting the relationships (Wickham and Winston 2014).

Principal Component Analysis (PCA) was employed to visualize differences in forest composition among the 10 forest sites. Patch utilization, browsing intensity and light availability were not used to construct the PCA, but later included as supplementary variables to show how they are associated with the PCA axes. A factor analysis was conducted to rank the correlation of the variables with the PCA axes. The package “FactorMineR, version 2.3” was used for the multivariate analysis and the package “factoextra, version 1.0.7” for plotting the graph (Lê et al. 2008; Kassambara and Mundt 2017).

An additional LMM analysis was conducted to identify how forest composition responds to herbivores (red and roe deer combined). In a first model we set tree diversity as a response and deer patch utilization, browsing intensity and light availability as fixed factors, forest site was used as a random factor to control for the nested design. In a second and third model, the same procedure was then repeated, but this time exchanging PCA Dim1 and Dim2 scores as response variables.

## Results

### Patch utilization and browsing intensity

**Fig. 3:**
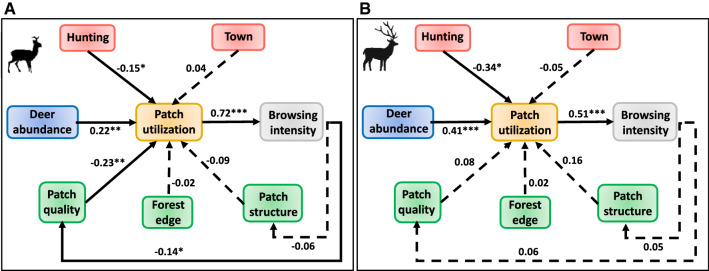
Path model showing how top-down, bottom-up and deer characteristics explain variation in patch utilization by roe deer (**A**) and red deer (**B**) in ten forest sites in the Netherlands. Variables are presented in squares and grouped by colour (i.e., red = top-down, green = bottom-up, purple = deer characteristics, yellow = patch utilization, grey = browsing), solid lines represent significant and dashed lines non-significant relationships. Numbers next to lines are the standardized coefficient of the relationships and an asterisk represents significance level: ***(*P* < 0.001), **(*P* < 0.01) and *(*P* < 0.05)

Across the ten forest sites, patch utilization presented a great variation among and within sites, providing an ample gradient for the core analysis of this study (Table 1, Electronic Supplemental Material A2–3). The results of the LMM indicated that patch utilization by roe deer decreased with hunting intensity (*β* = − 0.15, *p* = 0.05, Fig. 3A) and patch quality (*β* = − 0.23, *p* = 0.004), and increased with the landscape abundance of roe deer (*β* = 0.22, *p* = 0.007). Patch utilization was not significantly related to the distance to town, distance to forest edge, or patch structure (*p* > 0.21 in all cases). Browsing intensity increased with patch utilization (*β* = 0.72, *p* < 0.001) and patch quality decreased with browsing intensity (*β* = − 0.14, *p* = 0.05).

Patch utilization by red deer decreased with hunting intensity (*β* = − 0.34, *p* = 0.03, Fig. 3B) and increased with landscape abundance of red deer (*β* = 0.41, *p* < 0.001). Patch utilization was not significantly related to distance to town, distance to forest edge, patch quality or patch structure (*p* > 0.40 in all cases). Browsing intensity increased with patch utilization (*β* = 0.51, *p* < 0.001).

### Forest recruitment

**Fig. 4:**
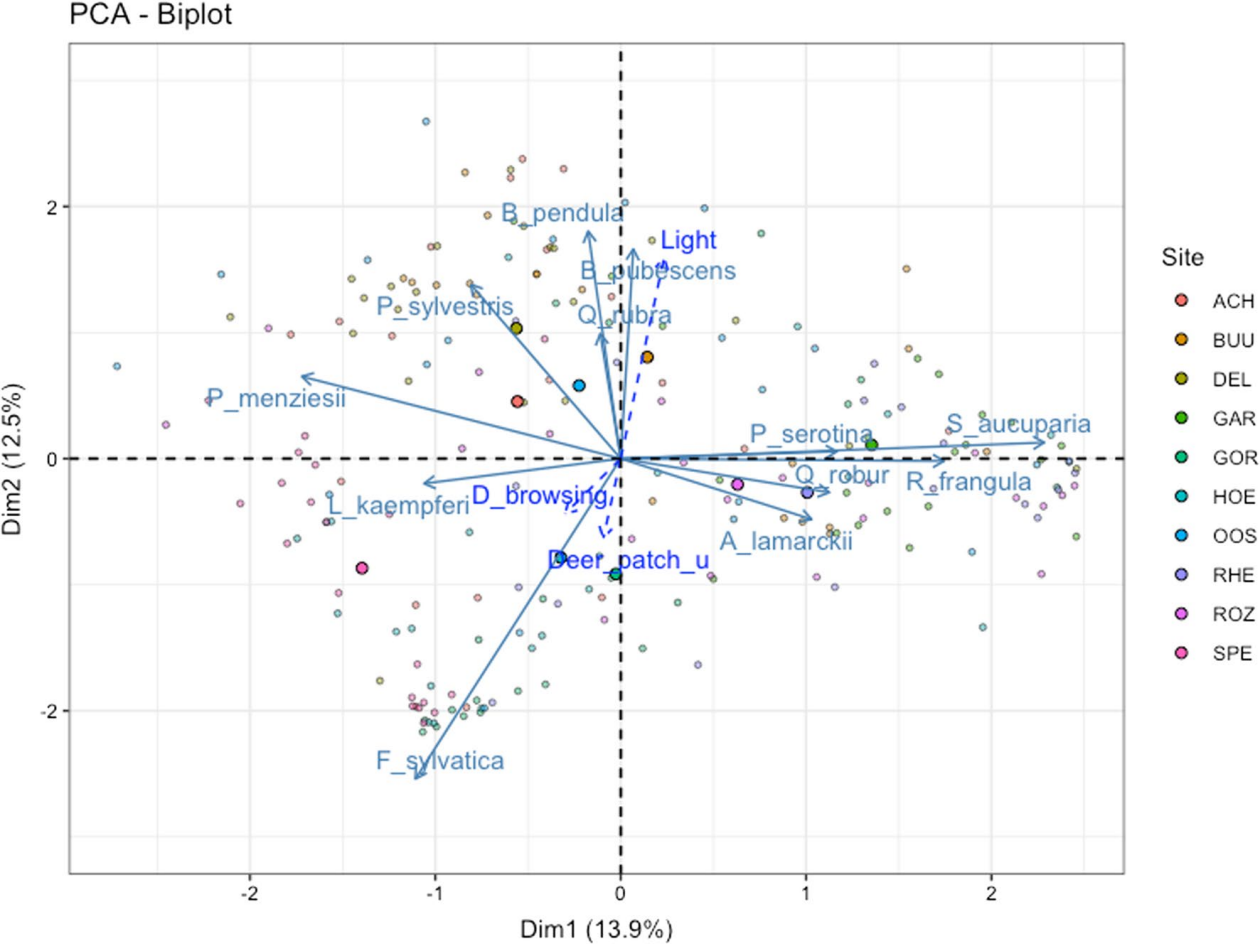
Principal Component Analysis for sapling (10–250 cm in height) surveyed across the vegetation sub-plots in all ten forest sites. Axis 1 (Dim1) explained 13.9% of the variation and axis 2 (Dim2) explained 12.5%. Sapling variables in light turquois represent the percentage of individuals per species in each sub-plot (*N* = 50 individuals). Superimposed variables in blue represent light availability (%), patch utilization (ind s m^−2^ d^−1^) and browsing (obs m^−2^ d^−1^) by roe and red deer combined. Small, coloured circles indicate individual vegetation sub-plots grouped by forest site. Large colour circles represent the average value per site

**Fig. 5:**
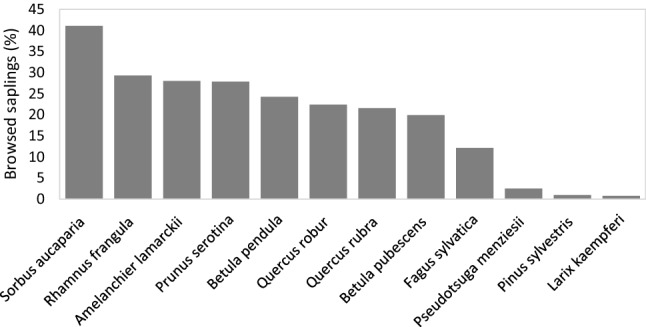
Representation of the total percentage of browsed saplings discriminated by species across the ten forest sites in the Veluwe, the Netherlands. The average number of assessed trees per species = 1630 (range 402–4394)

When all sapling species surveyed across the ten forest sites were plotted in a PCA, the first two axes explained 26.4% of the variation (Fig. 4). Dimension 1 (Dim1) explained 13.9% and was associated with a high percentage of *Sorbus aucuparia, Rhamnus frangula, Prunus serotina, Quercus robur* and *Amelanchier lamarckii* on the right side of the graph, whereas *Pinus sylvestris, Larix kaempferi, Fagus sylvatica* and *Pseudotsuga menziesii* were more abundant on the left side (Electronic Supplemental Material A4). Dim2 explained 12.5% of the variation and was associated with a high percentage of *Betula pendula, Betula pubescens, Pinus sylvestris, Quercus rubra* and *Pseudotsuga menziesii* on the upper part of the graph, whereas *Amelanchier lamarckii* and *Fagus sylvatica* were more abundant on the lower part. Only light availability (*r* = 0.49, *p* < 0.001) and merged roe deer and red deer patch utilization (*r* = − 0.19, *p* = 0.006) were correlated with Dim2. This suggests that Dim2 reflects palatability for red deer and roe deer. Browsing level varied across tree species, ranging from as much as 41% for *S. aucuparia* and 29% for *R. frangula* to less than 2.5% for the three conifer species: *P. menziesii*, *P. sylvestris,* and *L. kaempferi* (Fig. 5).

**Fig. 6:**
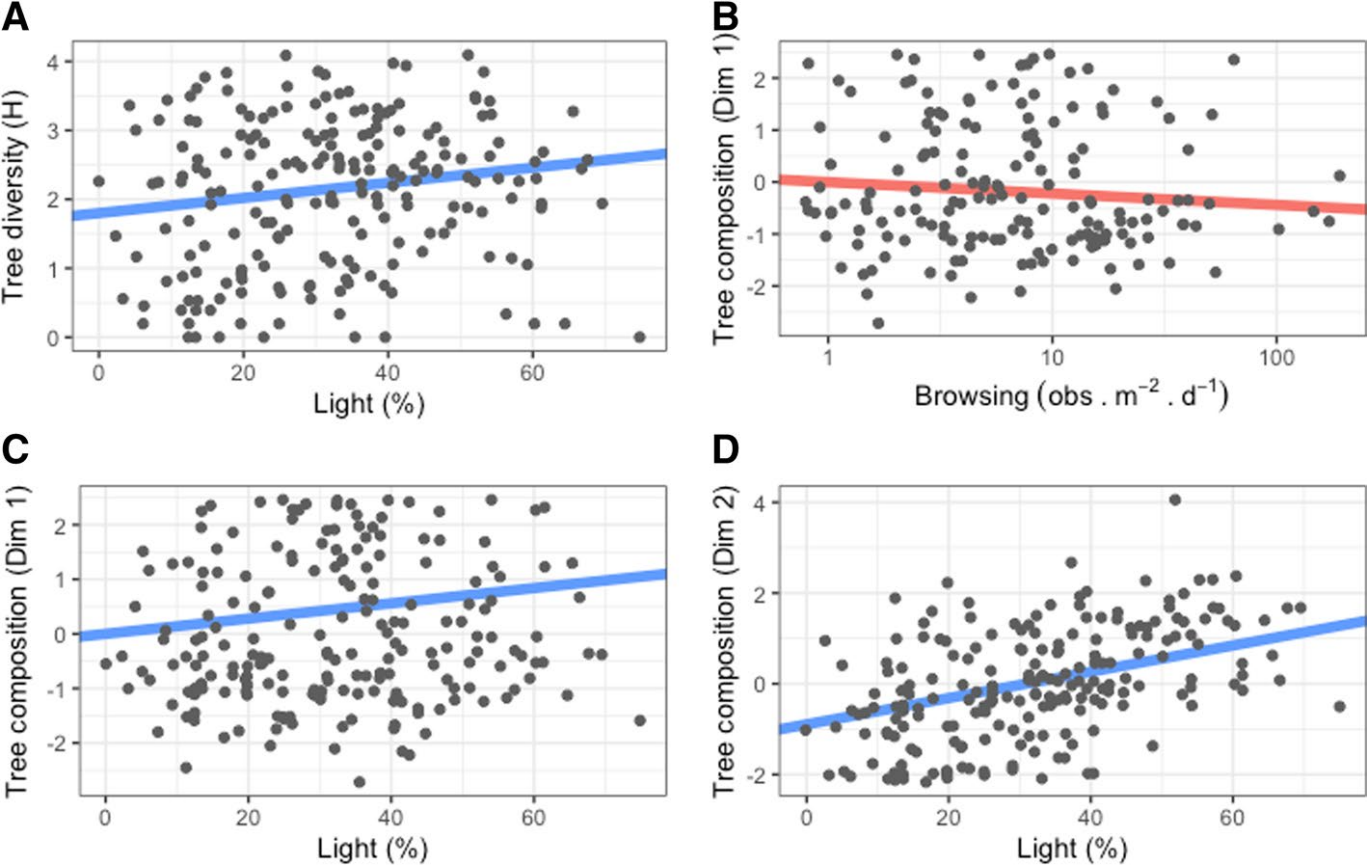
Linear Mixed Model fits related to forest recruitment as a response to light in the understory and herbivory by two deer species. Tree diversity (Shannon ‘H’) is set as response in panel **A**. Tree composition represents the values extracted from the PCA Dim1 and Dim2 and are set as responses in panels **B**–**D**. Regression lines represent absolute and not standardized coefficients to facilitate visualization. See Electronic Supplemental Material A5 for confidence intervals

The results of the LMM indicate that forest recruitment is explained mostly by light availability and partly by deer browsing (*β* = − 0.22, Table 2, Fig. 6B, Electronic Supplemental Material 5A). Specifically, light availability was stronger at explaining tree composition (*β*_Dim1_ = 0.18 and Fig. 6C, *β*_Dim2_ = 0.38 and Fig. 6D) than tree diversity (*β* = 0.16, Fig. 6A).

**Table 2:** Linear mixed models results

Response	*R*^2^ marginal	*R*^2^ conditional	P. utilization (*B*)	Browsing (*B*)	Light (*B*)
Tree diversity H	0.05	0.24	− 0.06	− 0.08	0.16*
Tree composition Dim1	0.04	0.41	0.14	− 0.22*	0.18**
Tree composition Dim2	0.17	0.37	− 0.12	0.03	0.38*

Response of tree diversity (Shannon ‘H’) and tree composition (Dimension 1 and 2) to patch utilization (ind s  m^−2^ d^−1^), browsing intensity (obs m^−2^ d^−1^) and light in the understory (%). Patch utilization and browsing intensity combine the values of red deer and roe deer. Standardized regression coefficients are presented (*B*) and see Electronic Supplemental Material A5 for confidence intervals. Significance is presented in bold and with an asterisk: ***(*P* < 0.001), **(*P* < 0.01) and *(*P* < 0.05)

## Discussion

This study aimed to explain large differences that exist in patch utilization by two deer species and forest recruitment in ten forests across the Veluwe, the Netherlands and assess how these are related. Results from our novel network of camera traps paired to vegetation plots confirm (Smith et al. 2020) the existence of top-down and bottom-up forces simultaneously affecting patch utilization as stated by the Optimal Foraging Theory (Hairston et al. 1960; Wilkinson and Sherratt 2016). Yet, this study suggests that the co-occurrence of these factors in modulating patch utilization are species specific, even when roe and red deer belong to the same guild and inhabit the same area. Variation in patch utilization increased browsing intensity, which in turn decreased patch quality, from a mix of broadleaf and conifer trees to mainly conifers.

### Patch utilization is equally determined by top-down, bottom-up forces and deer abundance

Patch utilization by roe deer was explained by top-down, bottom-up forces and landscape abundance of roe deer, which all had a similarly strong effect. Hunting intensity led to a decrease in patch utilization (*β* = − 0.15) probably by numerically reducing deer abundance and altering movement, browsing and vigilance rates (Ciuti et al. 2012). Surprisingly, patch quality also reduced roe deer patch utilization (*β* = − 0.23), perhaps because deer need less time to secure their food intake in patches which have higher proportion of broadleaf species compared to a situation where only a few broadleaf saplings are scattered in a conifer dominated forest stand. Patch utilization was positively explained by landscape abundance of roe deer (*β* = 0.22) which is in line with our prediction that deer find safety-in-numbers.

Bottom-up forces as predicted did not explain patch utilization by red deer since this is a mix feeder and hence, part of the time red deer spend grazing and resting in open environments. What we did expect and found is that hunting (*β* = − 0.34) and landscape abundance of red deer (*β* = 0.41) explained patch utilization in opposing ways since this species negatively responds to hunting by human and because this is a social species that benefits from living in groups (Georgii 1981; Lone et al. 2015). In line with our results, a companion study found that population size of red deer indeed positively explained variation in daily activity levels and spread (Ramirez et al. 2021c). Additionally, we expected that distance to forest edge would explain patch utilization as these areas are in the transition between forests and meadows and have therefore, a higher presence of herbaceous plants and a higher production of fresh tree leaves (Murcia 1995). The lack of relationship can be explained by the intensity which this area is used by recreationalists that are only allowed to use existing trails, shifting the perception of which areas are safe and which are risky.

Having both top-down and bottom-up forces simultaneously controlling patch utilization by deer are of great importance for promoting habitat heterogeneity (Hairston et al. 1960). A balance of top-down and bottom-up forces leads to an adequate browsing level that promotes habitat heterogeneity by modifying the landscape and creating opportunities for rare species. On the contrary, homogenous habitats are fostered when top-down and bottom-up forces are unbalanced, leading to a situation in which a system either lacks browsing or suffers of overbrowsing.

### Browsing intensity varies with ungulate feeding type

Patch utilization by roe and red deer results in a different browsing intensity. For roe deer and red deer, browsing intensity increases linearly with patch utilization, yet roe deer browsing (*β* = 0.72) presents a steeper response than red deer browsing (*β* = 0.51). This is explained by the fact that roe deer by being a strict browser and a forest species, browsing events are distributed in the same patch over long periods of time (Hofmann 1989; Tufto et al. 1996; Ramirez et al. 2018), which ultimately reduces the quality of the patch (*β* = − 0.14). Whereas browsing events by red deer will probably occur in shorter periods of time since this species is a mix feeder and spends part of the time in grasslands (Hofmann 1989), meaning that once they finish browsing, red deer move to open areas to graze and rest.

### Forest composition responds differentially to deer activity and light

Our field method does not have the power to distinguish how trees differentially respond to both deer species and thus, we can only test how saplings respond to the combined effect of roe deer and red deer. Thus, roe deer and red deer showed a weak increase in patch utilization in sapling communities dominated by *F. sylvatica* and a reduced patch utilization in sapling communities dominated by *B. pendula*, *B. pubescens, P. sylvestris*, *Q. rubra* and *P. menziesii* (Electronic Supplemental Material A4). This suggests that deer spend less time in palatable tree communities where they get more energy from readily available nutrients and hide in denser forest stand rather than in more open understory shrub stands. Interestingly, the positive association between *F. sylvatica* with deer patch utilization is explained by the tolerance to browsing by this tree species (Vandenberghe et al. 2008).

When assessing the total percentage of browsed saplings discriminated by tree species, deer have a strong preference for broadleaf species (browsed sapling ranged between 12 and 41% per species) over conifers (browsed saplings ranged between 0.8 and 2.5% per species). Selective herbivory often leads to changes in forest composition. Tree diversity increased as a response to light in the understory (*β* = 0.16). When assessing the responses of the PCA axes we found that tree composition along Dim1 was negatively explained by deer browsing (*β* = − 0.22) and positively explained by light (*β* = 0.18), while tree composition along Dim2 was positively explained by light (*β* = 0.38) and not by deer activity. Suggesting that abiotic conditions followed by herbivory are important factors shaping forest recruitment in these forests (Kuijper et al. 2010).

### Research outlook

We found that top-down and deer population abundance have similar, simultaneous and strong effects on both red and roe deer, but only roe deer were affected by bottom-up forces. The lack of relationship between red deer and bottom-up forces might have been an artifact of the chosen variables as they do not capture the full breadth of how the landscape matrix shapes behavioural responses of red deer. The Veluwe is the largest continuous forest in the Netherlands and attracts millions of recreationists every year. On-site, people engage in several activities that disturb the environment and because of the complexity of the interactions, it is not entirely understood how these activities affect wildlife. The most common are nature watchers, athletes and recreationalists. By scaling-up the survey design it will be possible to have a wider understanding of how population size of deer, top-down and bottom-up forces regulate patch utilization by a strict browser and a mix feeder (Ramirez 2021). This can be achieved by including all habitat types (i.e., forest, heath and grassland), quantifying number of visitors per area and applying a longitudinal approach that captures shifts in patch utilization by deer as a response to seasonality.

## Conclusion

We found that top-down and deer population abundance have similar, simultaneous and strong effects on both red and roe deer, but only roe deer were affected by bottom-up forces. Suggesting that the time roe deer spend in a patch is adequate for not increasing or decreasing the recruitment heterogeneity of these forests. Yet, light availability proved to be the most important factor in shaping forest composition and diversity. The fact that these results are species specific, even when species live in the same area and belong to the same animal guild, confirms that roe deer and red deer differentially experience, interact with and shape their surrounding landscape.

## Supplementary Information

Below is the link to the electronic supplementary material.

Supplementary file1 (DOCX 668 KB)

## Data Availability

Data used in the study are available from the corresponding author on reasonable request.

## Abbreviations

LMM: Linear mixed models
PCA: Principal component analysis

## References

[CR1] Apollonio M (1989). Lekking in fallow deer: just a matter of density?. Ethol Ecol Evol.

[CR2] Bates D, Mächler M, Bolker B, Walker S (2015). Fitting linear mixed-effects models using lme4. J Stat Softw.

[CR3] Baudry O, Charmetant C, Collet C, Ponette Q (2014). Estimating light climate in forest with the convex densiometer: operator effect, geometry and relation to diffuse light. Eur J for Res.

[CR4] Beauchamp G, Ruxton GD (2003). Changes in vigilance with group size under scramble competition. Am Nat.

[CR5] Bednekoff PA, Lima SL (1998). Re–examining safety in numbers: interactions between risk dilution and collective detection depend upon predator targeting behaviour. Proc R Soc Lond B Biol Sci.

[CR6] Brown JS (1988). Patch use as an indicator of habitat preference, predation risk, and competition. Behav Ecol Sociobiol.

[CR7] Brown JS, Laundré JW, Gurung M (1999). The ecology of fear: optimal foraging, game theory, and trophic interactions. J Mammal.

[CR8] Bubnicki JW, Churski M, Schmidt K (2019). Linking spatial patterns of terrestrial herbivore community structure to trophic interactions. Elife.

[CR9] Callan R, Nibbelink NP, Rooney TP (2013). Recolonizing wolves trigger a trophic cascade in Wisconsin (USA). J Ecol.

[CR10] Casaer J, Milotic T, Liefting Y (2019). Agouti: a platform for processing and archiving of camera trap images. Biodivers Inf Sci Stand.

[CR11] Charnov EL (1976). Optimal foraging, the marginal value theorem. Theor Popul Biol.

[CR12] China V, Kotler BP, Shefer N (2008). Density-dependent habitat and patch use in gerbils: consequences of safety in numbers?. Isr J Ecol Evol.

[CR13] Ciuti S, Northrup JM, Muhly TB (2012). Effects of humans on behaviour of wildlife exceed those of natural predators in a landscape of fear. PLoS ONE.

[CR14] Côté SD, Rooney TP, Tremblay J-P (2004). Ecological impacts of deer overabundance. Annu Rev Ecol Evol Syst.

[CR15] Cromsigt JPGM, Kuijper DPJ, Adam M (2013). Hunting for fear: innovating management of human–wildlife conflicts. J Appl Ecol.

[CR16] Davalos A, Simpson E, Nuzzo V, Blossey B (2015). Non-consumptive effects of native deer on introduced earthworm abundance. Ecosystems.

[CR17] Estes JA, Terborgh J, Brashares JS (2011). Trophic downgrading of planet Earth. Science (1979).

[CR18] Eycott AE, Watkinson AR, Hemami MR, Dolman PM (2007). The dispersal of vascular plants in a forest mosaic by a guild of mammalian herbivores. Oecologia.

[CR19] Fryxell JM (1991). Forage quality and aggregation by large herbivores. Am Nat.

[CR20] Gaynor KM, Hojnowski CE, Carter NH, Brashares JS (2018). The influence of human disturbance on wildlife nocturnality. Science (1979).

[CR21] Georgii B (1981). Activity patterns of female red deer (*Cervus elaphus* L.) in the Alps. Oecologia.

[CR22] Gill RMA (1992). A review of damage by mammals in north temperate forests: 1. Deer. Forestry.

[CR23] Gill RMA (1992). A review of damage by mammals in north temperate forests: 3. Impact on trees and forests. Forestry.

[CR24] Gill RMA, Beardall V (2001). The impact of deer on woodlands: the effects of browsing and seed dispersal on vegetation structure and composition. Forestry.

[CR25] Gill RMA, Morgan G (2010). The effects of varying deer density on natural regeneration in woodlands in lowland Britain. Forestry.

[CR26] Hager MC, Helfman GS (1991). Safety in numbers: shoal size choice by minnows under predatory threat. Behav Ecol Sociobiol.

[CR27] Hairston NG, Smith FE, Slobodkin LB (1960). Community structure, population control, and competition. Am Nat.

[CR28] Hamilton WD (1971). Geometry for the selfish herd. J Theor Biol.

[CR29] Hofmann RR (1989). Evolutionary steps of ecophysiological adaptation and diversification of ruminants: a comparative view of their digestive system. Oecologia.

[CR30] Kassambara A, Mundt F (2017) Package factoextra: extract and visualize the results of multivariate data analyses. R Package Version 1,0,7

[CR31] Kjellander P, Hewison AJM, Liberg O (2004). Experimental evidence for density-dependence of home-range size in roe deer (*Capreolus capreolus* L.): a comparison of two long-term studies. Oecologia.

[CR32] Kuijper DPJ, Cromsigt J, Jedrzejewska B (2010). Bottom-up versus top-down control of tree regeneration in the Bialowieza Primeval Forest, Poland. J Ecol.

[CR33] Kuijper DPJ, De Kleine C, Churski M (2013). Landscape of fear in Europe: wolves affect spatial patterns of ungulate browsing in Białowieża Primeval Forest, Poland. Ecography.

[CR34] Kuiters AT, Slim PA (2002). Regeneration of mixed deciduous forest in a Dutch forest-heathland, following a reduction of ungulate densities. Biol Conserv.

[CR35] Kuiters AT, Mohren GMJ, van Wieren SE (1996). Ungulates in temperate forest ecosystems. For Ecol Manage.

[CR36] Lê S, Josse J, Husson F (2008). FactoMineR: an R package for multivariate analysis. J Stat Softw.

[CR37] Lemmon PE (1956). A spherical densiometer for estimating forest overstory density. For Sci.

[CR38] Lima SL (1995). Collective detection of predatory attack by social foragers: fraught with ambiguity?. Anim Behav.

[CR39] Lima SL (1995). Back to the basics of anti-predatory vigilance: the group-size effect. Anim Behav.

[CR40] Lima SL (1998). Nonlethal effects in the ecology of predator-prey interactions. Bioscience.

[CR41] Lima SL, Dill LM (1990). Behavioral decisions made under the risk of predation: a review and prospectus. Can J Zool.

[CR42] Lima SL, Zollner PA (1996). Anti-predatory vigilance and the limits to collective detection: visual and spatial separation between foragers. Behav Ecol Sociobiol.

[CR43] Lone K, Loe LE, Meisingset EL (2015). An adaptive behavioural response to hunting: surviving male red deer shift habitat at the onset of the hunting season. Anim Behav.

[CR44] McNaughton SJ, Oesterheld M, Frank DA, Williams KJ (1989). Ecosystem-level patterns of primary productivity and herbivory in terrestrial habitats. Nature.

[CR45] Murcia C (1995). Edge effects in fragmented forests: implications for conservation. Trends Ecol Evol.

[CR46] Murray BD, Webster CR, Bump JK (2014). A migratory ungulate facilitates cross-boundary nitrogen transport in forested landscapes. Ecosystems.

[CR47] Nuttle T, Ristau TE, Royo AA (2014). Long-term biological legacies of herbivore density in a landscape-scale experiment: forest understoreys reflect past deer density treatments for at least 20 years. J Ecol.

[CR48] Pastor J, Naiman RJ (1992). Selective foraging and ecosystem processes in boreal forests. Am Nat.

[CR49] R Core Team (2013) R: A language and environment for statistical computing. R Foundation for Statistical Computing

[CR50] Ramirez JI (2021). Uncovering the different scales in deer–forest interactions. Ecol Evol.

[CR51] Ramirez JI, Jansen PA, Poorter L (2018). Effects of wild ungulates on the regeneration, structure and functioning of temperate forests: a semi-quantitative review. For Ecol Manage.

[CR52] Ramirez JI, Jansen PA, den Ouden J (2019). Long-term effects of wild ungulates on the structure, composition and succession of temperate forests. For Ecol Manage.

[CR53] Ramirez JI, Jansen PA, den Ouden J (2021). Temperate forests respond in a non-linear way to a population gradient of wild deer. Forestry: an Int J for Res.

[CR54] Ramirez JI, Jansen PA, den Ouden J (2021). Above- and below-ground cascading effects of wild ungulates in temperate forests. Ecosystems.

[CR55] Ramirez JI, Zwerts JA, van Kuijk M (2021). Density dependence of daily activity in three ungulate species. Ecol Evol.

[CR56] Rieucau G, Vickery WL, Doucet GJ, Laquerre B (2007). An innovative use of white-tailed deer (*Odocoileus virginianus*) foraging behaviour in impact studies. Can J Zool.

[CR57] Rieucau G, Vickery WL, Doucet GJ (2009). A patch use model to separate effects of foraging costs on giving-up densities: an experiment with white-tailed deer (*Odocoileus virginianus*). Behav Ecol Sociobiol.

[CR58] Seidel DP, Boyce MS (2015). Patch-use dynamics by a large herbivore. Mov Ecol.

[CR66] Shrader AM, Brown JS, Kerley GIH, Kotler BP (2008). Do free-ranging domestic goats show ‘landscapes of fear’? Patch use in response to habitat features and predator cues. J Arid Environ.

[CR59] Smith JA, Suraci JP, Hunter JS (2020). Zooming in on mechanistic predator–prey ecology: integrating camera traps with experimental methods to reveal the drivers of ecological interactions. J Anim Ecol.

[CR60] Tufto J, Andersen R, Linnell J (1996). Habitat use and ecological correlates of home range size in a small cervid: the roe deer. J Anim Ecol.

[CR61] Vandenberghe C, Freléchoux F, Buttler A (2008). The influence of competition from herbaceous vegetation and shade on simulated browsing tolerance of coniferous and deciduous saplings. Oikos.

[CR62] Vickery WL, Rieucau G, Doucet GJ (2011). Comparing habitat quality within and between environments using giving up densities: an example based on the winter habitat of white-tailed deer *Odocoileus virginianus*. Oikos.

[CR63] Wei W, Nie Y, Zhang Z (2015). Hunting bamboo: foraging patch selection and utilization by giant pandas and implications for conservation. Biol Conserv.

[CR64] Wickham H, Winston C (2014) Package ‘ggplot2.’ Create elegant data visualisations using the grammar of graphics 2:1–189

[CR65] Wilkinson DM, Sherratt TN (2016). Why is the world green? The interactions of top–down and bottom–up processes in terrestrial vegetation ecology. Plant Ecol Divers.

