# Supplementary material for: Top-down and bottom-up forces explain patch utilization by two deer species and forest recruitment
Source: Oecologia. 2022 Nov 24;201(1):229–40. doi: 10.1007/s00442-022-05292-8 (PMC9813088; doi:10.1007/s00442-022-05292-8)

Top-down and bottom-up forces explain patch utilization by two deer species and forest recruitment

*J. Ignacio Ramirez^1,2*^; Lourens Poorter^2^; Patrick A. Jansen^3,4^; Jan den Ouden^2^; Matthias Siewert^1^ & Johan Olofsson^1^*

**Affiliations**

*^1^Department of Ecology and Environmental Sciences, Umeå University, Umeå, Sweden.*

*^2^Forest Ecology and Forest Management Group, Wageningen University & Research, Wageningen, The Netherlands.*

*^3^Wildlife Ecology and Conservation Group, Wageningen University & Research, Wageningen, The Netherlands.*

*^4^Smithsonian Tropical Research Institute, Balboa, Ancon, Panama.*

*Corresponding author: [juanignacio.ramirez@icloud.com](mailto:juanignacio.ramirez@icloud.com) / [*+46 90 786 75 92*](tel:+46907867592)

**Electronic Supplemental Material**

*A1. Linear Mixed Model fits for the relationship between roe deer and red deer patch utilization. No significant (dashed line) relationship was found: R^2^marginal=0.05, R^2^conditional=0.06, β=0.009 & p value=0.68.*


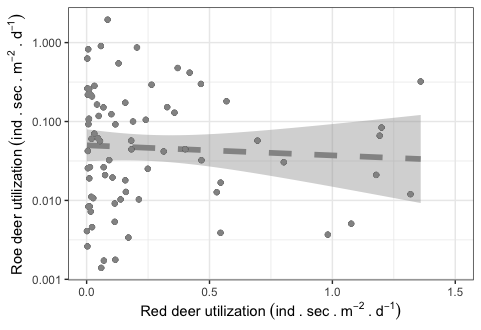


*A2. Map depicting the average patch utilization by roe deer (A) and red deer (B) across the ten forest sites. The size of the bubble indicates the utilization level for the specific forest site. Name of sites are presented in Fig. 1.*


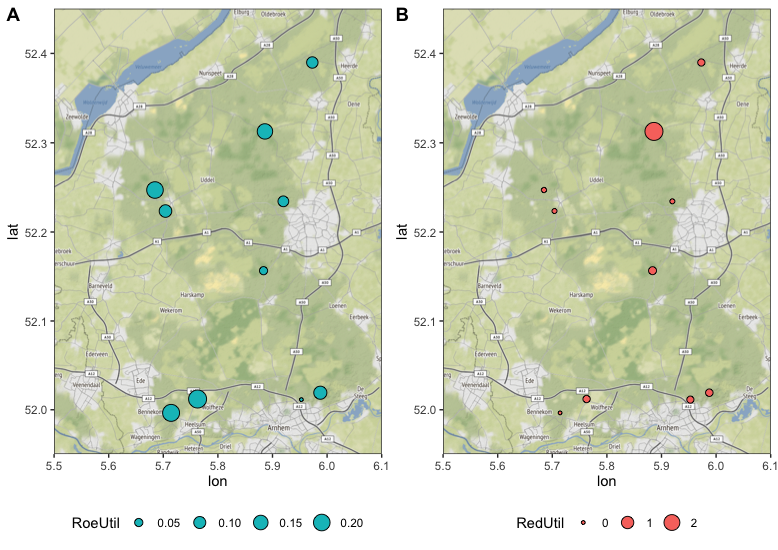


*A3. Patch utilization by roe deer (A) and red deer (B) for camera placements in each of the ten forest sites. The x-axis is the longitude (lon) and the y-axis the latitude (lat). The size of the bubble indicates the utilization level for the camera placement. Names of sites are presented in Fig. 1.*


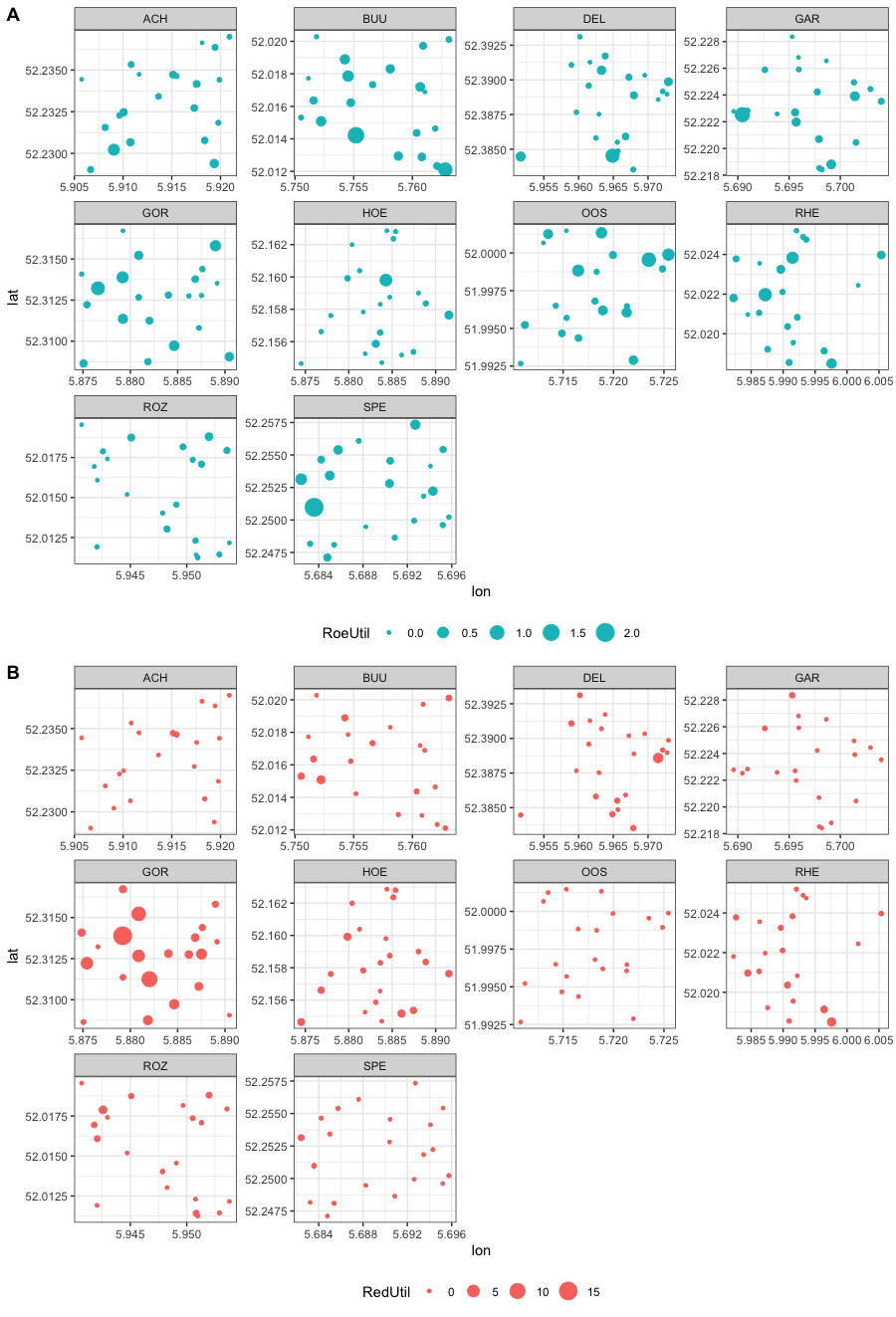


*A4. Variables that are the most characteristic according to each PCA axis obtained by a Factorial Analysis.*

| Variable | Correlation | p value |
| --- | --- | --- |
| *Dim 1* | |  |
| S_aucuparia | 0.70 | 9.9E-31 |
| R_frangula | 0.53 | 3.6E-16 |
| P_serotina | 0.36 | 2.0E-07 |
| Q_robur | 0.34 | 5.6E-07 |
| A_lamarckii | 0.31 | 5.2E-06 |
| P_sylvestris | -0.25 | 4.0E-04 |
| L_kaempferi | -0.32 | 2.6E-06 |
| F_sylvatica | -0.34 | 8.7E-07 |
| P_menziesii | -0.52 | 1.1E-15 |
| *Dim 2* | |  |
| B_pendula | 0.55 | 2.2E-17 |
| B_pubescens | 0.51 | 1.3E-14 |
| Light | 0.49 | 2.4E-13 |
| P_sylvestris | 0.42 | 4.2E-10 |
| Q_rubra | 0.30 | 1.2E-05 |
| P_menziesii | 0.20 | 4.5E-03 |
| A_lamarckii | -0.15 | 3.7E-02 |
| Deer_patch_u | -0.19 | 6.0E-03 |
| F_sylvatica | -0.78 | 9.8E-42 |

*A5. Linear Mixed Model fits related to forest recruitment as a response to light in the understory, patch utilization and herbivory by two deer species. Tree diversity (Shannon ‘H’) is set as response in panel A. Tree composition represents the values extracted from the PCA Dim1 and Dim2 and are set as responses in panels B & C. Models are accompanied by their standardized coefficients of the predictors, confidence intervals and p values represented by an asterisk (*). Significance levels: ∗∗∗(P<0.001), ∗∗(P<0.01) and ∗(P<0.05).*


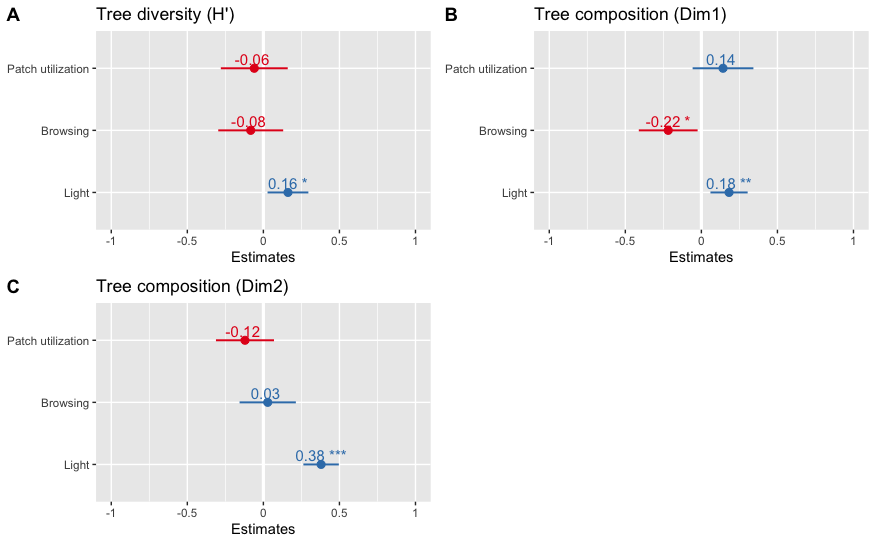

Supplement: Supplementary file 1 — Supplementary file1 (DOCX 668 KB) [file 442_2022_5292_MOESM1_ESM.docx]
